# Access to Maternal Health Services During the COVID-19 Pandemic: Experiences of Indigent Mothers and Health Care Providers in Kilifi County, Kenya

**DOI:** 10.3389/fsoc.2021.613042

**Published:** 2021-04-07

**Authors:** Stephen Okumu Ombere

**Affiliations:** Department of Sociology and Anthropology, Maseno University, Maseno-Kisumu, Kenya

**Keywords:** coronavirus, COVID-19, Kilifi county, maternal Health Services, traditional midwives

## Abstract

COVID-19 has spread rapidly in Kenya and has not spared pregnant women. Evidence from Kenya shows that during the COVID-19 pandemic, health systems have been either stressed to their maximum capacity or are becoming overwhelmed. However, the population is advised not to attend hospital unless strictly necessary, and this advice seems to apply to all, including expectant mothers. There is a dearth of information on how poor expectant mothers with low bargaining power cope during COVID-19 in Kenya, which this study addresses for those in Kilifi County. This rapid qualitative study draws data from an extensive literature review and from interviews with 12 purposively selected mothers who were either expectant or had newborn babies during the pandemic in Kilifi County. Five matrons-in-charge of maternal health services and four traditional birth attendants were also interviewed via mobile phone. Data were analyzed thematically and are presented in a textual description. It emerged that expectant mothers feared attending hospitals for perinatal care due to the possibility of contracting COVID-19. Therefore, there was an increase in home deliveries with the assistance of traditional birth attendants (TBAs)/traditional midwives, who were also overwhelmed with women who sought their services. Since most causes of maternal morbidity and mortality can be prevented by prompt, suitable treatment by qualified health practitioners, the health officials interviewed recommended training and integration of TBAs in emergency healthcare responses to help during crises in MHS because they are trusted by their local communities. Notably, such integration of traditional midwives should be supported and should also include additional training and monetary incentives.

## Introduction: Background and Context

Maternal health remains a challenge in low-resource countries. The numbers of women dying every year from maternity-related causes have remained high in such countries despite various efforts to bring them down (World Health Organization (WHO), 2019; Otieno et al., 2020). Pregnancy, childbirth, and postnatal states are a critical period in a woman's life; her health during this phase is known as maternal health. Most potential maternal morbidity and mortality can be prevented when prompt, suitable treatment is provided by qualified health practitioners, often referred to as “skilled birth attendants” (World Health Organization (WHO), 2018). Scientists continue to investigate the coronavirus and COVID-19, but little is yet known about the maternal and fetal birth outcomes of infected women. The world population has been waiting for answers and remains alert about the pandemic's progress. COVID-19 is still relatively new to humans, and only limited scientific evidence is available to identify its impact on sexual and reproductive health (SRH) (Tang et al., 2020).

Maternal health services and other sexual and reproductive health care such as family planning, emergency contraception, treatment of sexually transmitted diseases, post-abortion care, and, where legal, safe abortion services to the full extent of the law need to remain available as core health services. Early data from the United Nations Population Fund (UNFPA) suggests a drop in facility-based care in many countries and projections of rising maternal mortality as results of COVID-19 (UNFPA, 2020). During pandemics, health systems all over the world are either stressed to their maximum capacity or anticipating becoming overwhelmed (Iyengar et al., 2015; McQuilkin et al., 2017). The COVID-19 pandemic has disrupted access to healthcare services in many countries, and states are implementing measures to curb its spread. The best ways to stop the transmission of COVID-19 infection, as other articles in this Special Issue demonstrate, are physical distancing, mask wearing, and constant washing of hands and of potentially contaminated clothing, shoes, and surfaces. In Kenya, as the government has intensified its efforts to contain the spread of the virus, particular health workers and facilities have been redirected to deal with COVID-19 cases, which means that other health services, including maternal health care, are no longer priorities as they should and must be. A recent study reported that Kenya lacks a robust pandemic emergency preparedness plan, as human and financial resources are inadequate to respond to emergencies. Although existing disaster responses and risk mitigation committees include stakeholders across different sectors, these positions are politically motivated and lack adequate technical support (Wangamati and Sundby, 2020).

Globally, the COVID-19 pandemic has had devastating effects on health care delivery systems for people of all ages, but pregnant women face particular challenges. Rocca-Ihenacho and Alonso (2020) reported that the pandemic is making it increasingly challenging to provide adequate maternity care worldwide. Even the movement of people seeking to access health care services has been restricted in many countries to prevent the spread of the virus. The pandemic has led to a complete stoppage of the import and export of many essential commodities among various countries, leading to a shortage of necessary items and affecting healthcare services badly, especially sexual and reproductive health care (Kumar, 2020). The population is advised not to attend hospital unless strictly necessary; this advice seems to apply to all, including healthy pregnant women and even those with complications (Rocca Ihenacho and Alonso 2020). Therefore, the direct and indirect effects of the COVID-19 response on pregnant women, newborn babies, young children, and adolescents are enormous.

Improvements in maternal and child health care are principal targets of the Sustainable Development Goals (SDGs) under Health Goal 3.1. This goal mainly focuses on reducing the global maternal mortality ratio (MMR—the number of maternal deaths per 100,000 live births) to less than 70/100,000 live births by 2030 (World Health Organization, 2015). Between 2000 and 2017, the MMR dropped by about 38% worldwide (World Health Organization (WHO), 2019). However, in 2017, approximately 810 women around the world died every day from preventable causes related to pregnancy and childbirth. Maternal health services (MHS), which include antenatal, labor and delivery, and postnatal care, can play a crucial role in preventing and/or treating maternal health problems (Pant et al., 2020). However, the "obstetric" population is vulnerable, as different stages of pregnancy involve multiple interactions with the healthcare system; therefore, assisting the childbearing population presents unique challenges during the coronavirus pandemic. Postpartum hemorrhage, maternal sepsis, preeclampsia, and premature rupture of the membranes are the most common COVID-19-induced adverse events reported among pregnant women (Chen et al., 2020).

The consequences of COVID-19 could even be catastrophic for maternal and newborn health (Pant et al., 2020). Before the emergence of COVID-19 in Kenya, high-quality and timely maternity healthcare services were unavailable, inaccessible, or unaffordable for millions of women around the world, most especially in low-resource countries. Now, restrictions on travel and gatherings, health facilities with limited infection prevention supplies and unreliable infection control practices, and disrupted health worker's routines threaten to exacerbate limited access to care and further negatively impact women's health.

Kenya introduced free maternity services (FMS) in all public hospitals in 2013 to encourage skilled care deliveries and provide financial risk protection and equitable access to MHS for poor and vulnerable populations. Since the introduction of FMS, Kenya has made remarkable progress towards reducing mortality rates and improving coverage of health services (Kenya National Bureau of Statistics (KNBS), 2015). Yet despite such successes, considerable inequities in health outcomes and in uptake of health services remain, disadvantaging the most vulnerable individuals (Kenya National Bureau of Statistics (KNBS), 2015). Kenya recorded an increase in the proportion of facility-based deliveries from 44% in 2008 to 61% in 2015 (Kenya National Bureau of Statistics (KNBS), 2015). This increase in skilled care deliveries has been partly attributed to the free maternity care policy introduced in June 2013 (Kenya National Bureau of Statistics (KNBS), 2015; Pyone et al., 2017).

The first case of COVID-19 was reported in Kenya on March 13, 2020 and, like many countries across the globe, the Kenyan government has implemented measures and interventions to curtail the spread of the virus and to mitigate the socio-economic effects of COVID-19 response. Some of these steps, such as the nationwide dusk-to-dawn curfew, have negatively impacted access to essential health services, particularly emergency obstetric and newborn care. The curfew has restricted people’s movements, in particular by rendering all means of transport unavailable from dusk till dawn, including those of expectant mothers who need healthcare services. Moreover, many healthcare workers and facilities have been redirected to deal with COVID-19 cases; thus resources are often diverted away from maternal health care, making it increasingly challenging to provide adequate maternity care in Kenya. And studies from low resource settings have reported that limited supply of personal protective equipment (PPE) exposes healthcare providers and mothers to increased risk for infections (Strong, 2018; Karkee and Morgan, 2020), as has been the case in Kenya.

Despite the availability of free maternity services in all public health facilities, some counties in Kenya have reported high maternal and perinatal morbidities and deaths because laboring women could not access emergency transport to health facilities due to limited or no movement and fear of police during the pandemic. As one of my interlocutors (see below), a hospital matron (a nurse-midwife head of shift) stated:

More expectant mothers could die due to complications of other ailments when we are focused on COVID-19. The government needs to direct all agencies, including the police and other law enforcing agencies to provide emergency transportation for expectant mothers.

Furthermore, fear of contracting the coronavirus has kept many women away from seeking ante- and postnatal care. Healthcare providers in Kenya have also minimized in-person contact with their patients. So far, there is reduced utilization of maternal and child health services across the country as compared to 2019. Evidence from prior outbreaks shows that this crisis could exact a massive toll on women and girls. Women are disproportionally represented in the health and social services sectors, increasing their risk of exposure to the disease (UNFPA, 2020). Therefore, there is a need to explore alternatives that women from poor resource settings may use to gain access to MHS during the COVID-19 pandemic. This article describes how indigent mothers from Kilifi County, Kenya have responded to and coped with the dramatic changes that have occurred in birth practices as a result of this pandemic, primarily by choosing perinatal care with traditional midwives.

## Methods

This article follows on from my PhD ethnographic fieldwork on local perceptions of social protection schemes in maternal health in Kenya, conducted between March–July 2016 and February–July 2017 (Ombere, 2018). “Social protection schemes” include free maternity services in all public health facilities and maternal vouchers in selected accredited public and private health facilities (These vouchers were no longer in supply during my fieldwork). After my fieldwork ended, I have occasionally been in touch with most participants, who were poor mothers, health workers, community health volunteers and traditional birth attendants (TBAs), better referred to as traditional midwives because they are regarded as such by their communities (Davis-Floyd 2018). Since March 2020, when the first case of COVID-19 was reported in Kenya, out of 40 mothers whom I followed during my ethnographic fieldwork in Kilifi County, I managed to reach 20 of them between June 13 and July 24, 2020. Data for this article were derived from the responses of 12 of these mothers who were either expectant or gave birth during the COVID-19 pandemic.

For this qualitative study, I also conducted phone interviews with five matrons (nurse-midwives who serve as department heads) in charge of maternal health services, whom I purposively selected from the two busiest health facilities that I had contact with during my initial fieldwork. And I interviewed four traditional midwives (locally called mkunga in the singular and wakunga in the plural), based on the roles they had previously played in referring expectant mothers to facilities for delivery. Most of these phone conversations were recorded and later transcribed. I teased out emerging themes for this article based on the overall objectives of this Special Issue on the impact of COVID-19 on both maternity care practices in various countries and on pregnant families' experiences of maternity care. All participants were informed about the nature of this study and only those who consented to the phone interviews participated. To maintain confidentiality and minimize the potential of identification of study participants, no identifying names have been used in reporting the study findings. Ethical approval was obtained from Maseno University Ethical Review Committee (Reference #MSU/DRPI/MUERC/00206/015). For this article, I also carried out an extensive literature review of research related to my subject matter.

## Findings: Fear of Infections, Lack of PPE, and an Increase in Traditional Midwife-Attended Births

### Fear of Infections and Lack of PPE in Hospitals

Steve, I tell you I cannot go to the hospital when I know very well I am going to get coronavirus. I know my friends who have been attending antenatal clinics who also fear going to the hospital. At least I know there is a mkunga who will assist me (Pregnant mother, expecting her seventh child)

I avoided going for the clinic since corona began. I had visited the hospital once and again, going to the hospital using a motorbike is still risky because I am among the groups at risk contracting the virus and I cannot tell the virus status of those who boarded the motorcycle. I will deliver at home (Pregnant mother, expecting her third child)

No need for risking. My child can miss all those clinics till that time they will say coronavirus is managed and is no longer there with us. I will just give my child healthy foods such as porridge and cow milk (A mother, supposed to go for postnatal care)

Fear of coronavirus has affected mothers differently. It is true most pregnant mothers and those that are supposed to attend clinics have avoided coming for routine clinics (Maternity matron, public health facility 02)

The excerpts above denote the recurring theme of fear of contracting the coronavirus. Pregnant and breastfeeding mothers expressed anxiety and worry about COVID-19; thus, a majority of them avoided going to antenatal and postnatal clinics. There was a general knowledge among pregnant women that they had low immunity and thus could easily get infected by the virus. Expectant mothers expressed their confidence in delivering with the assistance of traditional midwives in their community. Mothers who had delivered would not take their babies for postnatal check-ups due to fear, resulting in missed vaccinations and other possibly essential medicines.

For their part, due to the lack of adequate protective gear, health workers also expressed fear of treating people from the community, including those who visited clinics for ante- and postnatal care. These health workers noted that even though there are government guidelines on safety measures during COVID-19, the chances were very high that poor mothers from the villages could not afford even an ordinary mask or sanitizer, and thus could expose health workers to danger. As noted above, these health facility matrons also reported that few mothers were attending maternity clinics due to fear of contagion. The matrons feared that the number of maternal deaths could increase due to pregnancy-related complications during COVID-19. Two of them stated:

I am a human being, we don’t have enough protective gear. Thus, I fear handling people from the village including the expectant mothers. I cannot know who they interacted with and how they behaved outside there. Moreover, the women around here are very poor, and some cannot even afford the face masks (Maternity matron, public health facility 01)

We have few mothers reporting for the antenatal and postnatal clinics. Many children have not got their immunization. I know this is due to fear of COVID-19 (Maternity matron, public health facility 02)

### Increase in Traditional Midwife-Attended Births

The traditional midwives interviewed—who tend to be older women, as few younger women want to carry on their time-honored practices—reported that there was a rapid increase in the number of women whom they assisted in delivering due to various factors, including women’s fears of hospital infection and the reduction in the availability of primary healthcare services, including health facility deliveries. This increase was challenging for the traditional midwives to manage because almost all expectant mothers in the villages preferred giving birth in traditional midwives' homes, which have no beds to accommodate the expectant mothers. Usually there is a small hut for delivery in or near every traditional midwife’s home. Plastic polythene is spread on the mud floor for deliveries. Poor women prefer delivering at traditional midwives’ homes as those birthing spaces are often cleaner than their own homes. Another advantage is that they can pay in installments and sometimes work on the midwives’ farms after they have healed by way of payment. Thus birth with a traditional midwife is often cheaper than facility birth (see below).

#### Two Wakunga/Community Midwives Stated

Stephen, this virus has given me a lot of work, but again I am overwhelmed because all women from [Village Y] come to my home and you know I don't have beds here. They deliver, sit for an hour or so and their husbands collect them. I have a small hut special for assisting mothers to give birth. The floor is smeared with mud but I do spread a polythene bag then wash it after completing delivery. Women come here because I know them and they can always pay later or work on my farm after healing (Traditional midwife 002)

As the mkunga, I assist women daily. Okay, some women had complications and from my records, three babies died when I was trying to help the mothers deliver. They died because two women came late when the complication was too hard for me to handle, while one was a severe complication that I could not handle. I managed to remove the baby, but it was already dead (Traditional midwife 005)

This latter statement reveals the need for training traditional midwives in how to better handle birth complications.

Concomitantly with the rise in traditional midwife-attended births, the hospital matrons noted a decrease in hospital deliveries. As previously mentioned, many health facilities were closed because the health workers were reassigned to handle COVID-19 cases, preventing mothers from delivering there. However, health workers warned that although giving birth at home was an available option—still used pre-COVID by almost 40% of pregnant women—giving birth in a hospital was nevertheless the safest way because all complications could be handled in the health facility even during the pandemic. According to two matrons:

Women have been giving birth at home long before hospitals even existed. The problem comes in when there is a complication that needs doctors’ attention; we are likely to lose the mother and the child (Hospital matron 01)

It is true many health facilities were temporarily shut down and health workers reassigned duties to handle COVID-19 cases. It means that poor expectant mothers had to look for alternatives on where to deliver and definitely they went to TBAs (Hospital matron 02).

Mothers who participated in this study noted that they could not go against their husbands’ advice and delivering in traditional midwives’ homes was cheaper, which is why their husbands preferred this option. These mothers argued that wealthy pregnant women could go to private health facilities and also have the ability to bargain for what they want. Two mothers explained:

It is true we are delivering at the mkunga’s home during this coronavirus period. Giving birth there is cheaper because we can pay using different ways. Our husbands also have the final say, so after looking around we resort to the mkunga, which is a better option. (Mother from Village D)

I cannot go to a private health facility because I don’t have enough money. Private hospital is for the rich who know how to bargain and their money speaks for them, they don’t beg to get services. But during this coronavirus time it is very expensive to give birth at a private health facility (A mother from Village G)

These quotes beg the question: Why don’t these economically disadvantaged women use the free public hospitals for birth? The issue from these mothers’ perspective is that “free” maternity services are never truly free. Poor women, who have low bargaining power, are expected to pay some money before getting some essential services in public health facilities. For examples, they have to pay for laboratory services, antenatal care, and sometimes have to purchase any medicines prescribed. Thus to them, it is expensive to give birth in even a “free” public health facility, and private health facilities are completely beyond their reach.

To avert home births during COVID-19, health workers suggested that the government could direct all agencies, including the police and other law enforcement agencies, to provide emergency transportation to health facilities to minimize maternal deaths from complications occasioned by delay in seeking care during COVID-19 due to the curfew. However, some health workers argued that traditional midwives should be better trained in how to handle complications because the community trusts them and they can help avert maternal deaths during this or other crises. For example, one matron stated:

Traditional birth attendants have really helped many poor mothers in the villages. They are trusted by the mothers, I think there is need to always re-train these TBAs to avert maternal deaths due to complications during pandemics (Hospital matron 05).

Yet no such re-trainings have as yet been offered.

## Discussion and Analysis: Making Tough Choices that Leave Women out, and Incorporating Traditional Midwives

My study findings reflect how pregnant mothers from the poor socio-economic class in Kilifi County are vulnerable during COVID-19 in Kenya. Again, sexual and reproductive health care services are essential for any community and are usually neglected and seriously affected during epidemics and pandemics, leading to long-term adverse consequences (Kumar, 2020). Evidence from other studies shows that decisions made at every level of the response to the pandemic are resulting in women being further cut off from sexual and reproductive health services, threatening sharp rises in maternal and neonatal mortality (Phumaphi et al., 2020; Pollock et al., 2020). Now, this global pandemic is making a bad situation even worse, as some countries divert resources away from other essential services (Phumaphi et al., 2020). UN Women (2020) warned that the diversion of attention and critical resources away from the provisions of sexual and reproductive health services, including maternal health care, might result in aggravated maternal mortality and morbidity. Indeed, my matron interlocutors agreed that frontline providers were forced to make tough choices about which services are most important, and women were often left out. However, even before the COVID-19 pandemic, global progress towards the 2030 Every Woman Every Child (EWEC) Global Strategy for Women's, Children's and Adolescents' Health target to save the lives of women and children was already lagging by around 20%.

Findings from this study indicate that there is a likely increase in maternal and neonatal deaths during the COVID-19 pandemic in Kenya. This increase can be attributed to the effects of the pandemic, which have led to delays in accessing life-saving procedures such as cesareans, due to staff deployment and shortages, fear, and lack of infrastructure. This concurs with recent findings from other studies that also report an increase in maternal deaths globally due to COVID-19 (Hussein, 2020; Takemoto et al., 2020). Supporting my own findings, Kimani et al. (2020) also noted for Nairobi, Kenya that fear of contracting COVID-19 likely kept many women from attending reproductive health services. Relatedly, Delamou et al. (2017) showed that fear of infection at health facilities was also reported by women in Guinea during the recent Ebola epidemic there (see also Strong and Schwartz 2016).

The results of my study show that there was a decrease in the utilization of maternal health services among the childbearers in Kilifi County, including antenatal, labor and delivery, and postnatal services. This was occasioned by fear of contracting the virus and low bargaining power for access to better health care. My findings corroborate recent findings by Pant et al. (2020), who noted that decreased access and utilization of maternal health services could have dire consequences for both women and newborns. Pregnant and postpartum women are already at high risk of nutritional deficiency during the lockdown due to decreased supply of nutritious food. On top of that, when they are unable to have regular antenatal and postnatal services, they are deprived of the micronutrient supplements that they get from the clinics. In addition, without regular checkups, there are chances of certain danger signs going unidentified, which makes them vulnerable to complications related to pregnancy and childbirth. Health workers also reported that fear of COVID-19 transmission in hospital settings was widespread because of a scarcity of proper PPE in the health facilities. A study in Nepal (Karkee and Morgan, 2020) and another in Tanzania (Strong, 2018) also confirmed that scarcity of PPE and fear were some of the factors affecting women’s access to safe delivery, which is within their rights, by extending the well-known “three delays” in deciding to go to a health facility, in reaching it, and in receiving quality care once they arrive.

Although access to safe delivery care has long been acknowledged as an essential health service, many poor pregnant women in Kenya as a whole suddenly found themselves with fewer options for care as health facilities were converted into isolation wards. Wangamati and Sundby (2020) also noted that such changes led to confusion, as pregnant women and mothers did not know where to go to seek maternal health services. In this study, a majority of mothers resorted to giving birth in the homes of the traditional midwives in Kilifi, since most health facilities were temporarily shut down and health workers were reassigned to the COVID-19 crisis. It emerged that traditional midwives were valued by expectant mothers because they are well-respected, easily accessible during COVID-19, offer flexible payment modalities, understand and abide by local customs and traditions, and provide services that skilled birth attendants do not—such as pre- and postnatal massage and more compassionate care. These findings are corroborated by Byrne et al. (2016) and Ombere (2018), who reported much the same. However, the government of Kenya actively discourages TBA-supported births (Byrne et al., 2016), preferring that TBAs refer mothers to the nearest health facility. According to the health workers in this study, integration of traditional midwives during pandemics such as COVID-19 and other crises is necessary. This recommendation from the health workers concurs with findings from a recent study in Kenya by Kimani et al. (2020), who argued that integrating community health workers by expanding existing midwifery centers and creating new ones run by qualified midwives (“skilled birth attendants”) that are closer to or in rural communities could be a viable long-term plan that can reduce the burden on hospitals, and minimize infections and maternal deaths during pandemics such as COVID-19.

Given that studies from the Democratic Republic of Congo (Matendo et al., 2011) and Kenya (Mannah et al., 2014; Bucher et al., 2016) indicate that training TBAs averted maternal deaths, there is a need to re-define the roles and responsibilities of traditional midwives in maternal and neonatal health care during pandemics and in more normal times. Again, viable and culturally respectful TBA training programs must be developed and widely taught, especially given that fact that around 40% of Kenyan childbearers were still choosing to birth with TBAs pre-pandemic. As my interviews showed, traditional midwives themselves admit that, despite being trusted by their communities, there are complications they do not know how to handle. There is also the problem that if TBAs are only called on during crises, and not allowed to attend births under normal conditions, they may lose any skills they have gained during trainings due to lack of practice. Thus I and others strongly suggest that traditional midwives should be fully incorporated into the Kenyan maternity care system and facilitated to attend births in all circumstances. As in other countries, such as Nigeria, Somalia and Ghana (Pyone et al., 2014; Chukwuma et al., 2019; Haruna et al., 2019), Kenya can also provide monetary incentives to the traditional midwives for maternal services referrals and for attending births. Re-training wakunga will not only enhance their knowledge and skills in maternity care and referral mechanisms, but will also lead to greater community acceptance and client satisfaction (Smith et al., 2000; Haruna et al., 2019). For example, Dynes et al. (2013) and Buffington et al. (2021) have shown how TBAs can be trained in the safe administration of Cytotec/misoprostol to stop post-partum hemorrhages and to successfully deal with other birth complications.

## Conclusion: Future Recommendations

The COVID-19 pandemic is an exceptional event that took the world by disbelief. It has caused the interruption of health services on a global scale, including maternal health services. COVID-19 has spread rapidly in Kenya and has not spared pregnant women. Based on my study findings, I recommend that, as governments alter their healthcare systems to deal with COVID-19 or any future pandemic, they must also act urgently to ensure that mothers and newborns are still able to get the routine and emergency care they need. This includes ensuring that funds for pandemic response go toward efforts to ensure continuity of maternity care, with adequate funding for infection prevention and control supplies and sufficient PPE for maternity care providers, including TBAs. It also includes full training and integration of these traditional midwives—who, again, still attend 40% of Kenyan births—that facilitates them to practice both in normal times and those of crisis. Full integration for TBAs should include allowing them to enter healthcare facilities with their clients and remain with them throughout labor, delivery, and the postpartum period to provide culturally safe continuity of care (see Davis-Floyd, 2003). Referral pathways and transportation must be provided for obstetric emergencies, and hospitals need to be able to properly screen, isolate, and care for infected pregnant women. Guidelines specific to reproductive age and to individual pregnant women need to be developed and effectively communicated to women and to traditional midwives in their own languages. Moreover, motivating traditional midwives using monetary incentives can increase early antenatal and postnatal care use among mothers. In conclusion, again I stress that traditional midwives should not only be utilized in times of crisis, but also under normal circumstances, forming an integral part of Kenya’s maternity healthcare system. And for future research, I point to the need for longitudinal studies to explore the experiences of indigent mothers and healthcare providers around access and utilization of maternal health services during COVID-19 and other pandemics yet to come.

## Data Availability

The raw data supporting the conclusion of this article will be made available by the authors, without undue reservation.
